# The Role of Attachment in Refugees with Impaired Mental Health: A Systematic Review

**DOI:** 10.3390/brainsci15050495

**Published:** 2025-05-09

**Authors:** Thomas Egger, Anna Buchheim, Manuela Gander

**Affiliations:** 1 Institute of Psychology, University of Innsbruck, 6020 Innsbruck, Austria; anna.buchheim@uibk.ac.at (A.B.); manuela.gander@uibk.ac.at; 2 Refugio München, Rosenheimer Str. 38, 81669 Munich, Germany; 3 Universitätsklinik für Kinder- und Jugendpsychiatrie, Medizinische Universität Innsbruck, Anichstraße 35, 6020 Innsbruck, Austria

**Keywords:** attachment, refugees, mental health, PTSD, depression, anxiety, forced migration, adult attachment interview, ECR, reflective functioning, culture

## Abstract

Although the relationship between attachment and mental health has been widely studied, no systematic review has focused specifically on refugee populations. **Objectives:** This systematic review examines associations between attachment patterns and psychological distress in refugees—a population at elevated risk for mental health disorders due to forced displacement and trauma. **Methods:** Following PRISMA guidelines. we searched PubMed, PsycINFO, and Web of Science (last search: 5 October 2024). Studies were included if they examined the relationship between attachment and psychological distress or disorders in refugees, presented empirical data, were peer-reviewed, were published from 2004 onward in English, and met quality criteria based on CASP and JBI checklists. Studies were excluded if they did not focus on refugees, lacked empirical data, did not assess both attachment and psychological distress, were not peer-reviewed, or consisted of grey literature. A narrative synthesis was conducted. **Results:** Of 2.951 records, 11 studies with 1.319 participants met inclusion criteria. Five studies examined adults, four children, and two adolescents. Insecure and unresolved attachment were consistently linked to higher psychological distress, particularly PTSD, especially in adults. In children, insecure attachment was associated with parental mental health problems and dysfunctional parenting, whereas secure attachment buffered the effects of parental PTSD. **Discussion:** Limitations include small sample sizes, cultural and linguistic complexity, inconsistent definitions of “refugee”, and varied assessment methods. **Conclusions:** Attachment insecurity is strongly associated with psychological distress in refugees, mirroring patterns in Western clinical populations. Findings support the integration of attachment-informed approaches into refugee mental health care. Funding: This review was funded by the Köhler Stiftung and registered in PROSPERO (CRD42024590759).

## 1. Introduction

Numerous studies have investigated the effects of attachment and trauma in various contexts [1,2,3,4]. Although several publications have addressed the relevance of attachment theory in refugee contexts, no systematic review has specifically synthesized primary empirical studies—including those using qualitative or mixed-method designs—that examine both attachment and psychological distress in forcibly displaced populations. This review addresses that gap. Following Prisma-Guidelines [5], it aims to provide a focused synthesis of the existing empirical evidence in this area.

The present systematic review aims to close this gap by assessing the state of research on the links between attachment and mental illness in an increasingly relevant population: refugees. The growing body of research exploring the links between attachment and mental disorders [6], including depression [7], suicidal thoughts [8], OCD [9], or social anxiety [10] in adults underlines the relevance of this topic. Equally significant and widely studied is the role of attachment in child development and psychopathology. In childhood, the correlation between insecure attachment styles and externalizing behavioural problems [11] is apparent, particularly for the disorganized attachment pattern [12]. Unresolved attachment representations are overrepresented in all major clinical disorders. Adults with past or current experiences of abuse or post-traumatic stress disorder (PTSD) are therefore predominantly classified in the “unresolved” category [13].

### 1.1. Associations Between Attachment and Trauma

If adult attachment patterns are examined in relation to clinical symptoms, methodological implications must be considered. In adults, secure, insecure (preoccupied and dismissing), and unresolved attachment representations are classified as four distinct constructs in well-established categorical instruments such as the Adult Attachment Interview [14,15] and the Adult Attachment Projective Picture System [16]. In dimensional approaches, insecure adult attachment style is typically represented along two continuous scales: attachment anxiety and attachment avoidance. Notably, self-report questionnaires do not always include the fearful-avoidant or unresolved pattern as a distinct dimension [17]. Furthermore, inconsistencies in study quality and result reporting hinder clear conclusions regarding which measure best captures adult disorganized attachment [18]. If studies do not report on disorganized or unresolved attachment specifically but use only the mentioned insecure dimensions (anxious, avoidant), it can be assumed that fearful-avoidant attachment is at least partially represented within these insecure scales. Nevertheless, associations between trauma and attachment will be presented separately, beginning with studies that did not distinguish between insecure and unresolved attachment.

Secure attachment helps individuals to cope with trauma through adaptive strategies and supportive mental representations, whereas insecure attachment increases the risk for severe PTSD following trauma exposure [19]. With regard to posttraumatic symptoms, the type and timing of trauma play a crucial role in the development and manifestation of attachment security or insecurity. Interpersonal traumas—such as physical, sexual, or psychological assault—tend to have a greater negative impact on psychological functioning than non-interpersonal traumas, as they are more likely to activate the attachment insecurity system and disrupt behavioural, emotional, and relational responses [20]. Insecure attachment has a stronger impact on PTSD symptoms when trauma occurs in early childhood compared to adulthood. This may be explained by the fact that early traumatic experiences can severely interfere with the development of secure attachment patterns. As a result, essential skills for regulating negative emotions and coping with stress may be impaired, increasing vulnerability to PTSD. Specifically, attachment anxiety has been found to be a stronger predictor of PTSD symptoms following early childhood trauma than trauma in adulthood [1]. Higher attachment security is associated with a stronger therapeutic alliance, while attachment insecurity is linked to a weaker alliance [21]. Both anxious and avoidant attachment representations have been found to negatively correlate with the working alliance [22]. At the same time, attachment security tends to increase through therapeutic interventions, while attachment anxiety may decrease [6].

Studies that specifically include the fearful attachment style show strong evidence of its impact. A meta-analysis of 46 studies found that secure attachment is linked with lower PTSD symptoms following trauma, while insecure attachment is associated with higher PTSD symptoms, with the fearful attachment style showing the strongest association with PTSD symptoms [2]. Among clinical populations, the unresolved attachment classification—typically associated with trauma—is the most commonly observed [6]. In children and adolescents, significant correlations emerge as well: Secure attachment is negatively associated with posttraumatic stress symptoms, whereas insecure and disorganized attachment show positive associations [3]. An individual’s attachment style, shaped by trauma experiences, also affects how they bond with their children [23]. While one study found only a weak but significant link between a parental history of childhood maltreatment and child attachment insecurity—and no link to attachment disorganization [24]—a meta-analysis found that unresolved attachment is overrepresented among maltreating parents compared to non-maltreating ones. Insecure attachment is also linked to an increased risk of child maltreatment and greater potential for physical abuse [25]. Attachment has also been associated with somatic symptoms in individuals with traumatic childhood experiences. Insecure attachment predicted the presence or intensity of somatic symptoms, which have been examined in relation to DNA damage, obesity, functional neurological disorders, and somatization. Unresolved attachment was associated with metabolic syndrome and emotional abuse [26]. Insecure attachment increases vulnerability to developing PTSD. Attachment style may influence how distress is expressed: Individuals with a preoccupied attachment style tend to report heightened PTSD symptoms, whereas those with a dismissing style may underestimate symptoms and somatize them. Fearful or unresolved attachment has been associated with greater severity of PTSD symptoms than other attachment styles. Beliefs about the availability of social support and willingness to seek help also appear to vary by attachment style [4].

The strong association between unresolved attachment and PTSD underscores the importance of considering this attachment style in both psychopathological assessment and psychotherapeutic treatment. Attachment research plays a crucial role in understanding trauma-related disorders and should be integrated into clinical practice, particularly for individuals from diverse cultural backgrounds.

### 1.2. Mental Health in Refugees

The close connection between attachment and trauma becomes particularly relevant when considering the experiences of refugees. Asylum applications in Europe peaked in 2015 at over 1.2 million and, after a decline until 2020, have been rising again, surpassing 1 million in 2023 [27]. Since 2014, the global number of forcibly displaced people has been increasing, reaching a new high of 117.3 million in 2023. Similarly, the number of refugees, individuals in refugee-like situations, and other people in need of international protection has risen steadily since 2014, now totalling 37.4 million. The United States is the world’s largest recipient of new individual asylum applications (1.2 million), followed by Germany with 0.3 million. Among the greatest dangers faced by those fleeing are physical violence, theft, and imprisonment [28]. In addition to that, physical and mental disorders represent a considerable risk factor.

The most common mental disorders among refugees are depression and PTSD, with a prevalence of approximately 31.5% to 32% for depression and 31% to 31.5% for PTSD [29,30]. This means that among refugee adults, the prevalence of depression is seven times higher, and PTSD is four to five times higher than in the general population [30]. Among refugee children and adolescents, the most frequent disorders are PTSD (prevalence = 22.71%), anxiety disorders (15.77%), and depression (13.81%). These rates also far exceed those of non-refugee comparison groups [31]. Both children/adolescents and adults have significantly higher prevalence rates of anxiety, depression, and PTSD—whether diagnosed or self-reported—compared to non-refugee populations worldwide, including those living in conflict or war settings [32]. Migrants with previous exposure to violence also show a high prevalence of mental disorders [33]. Refugee children are in considerable danger of being affected by physical [34] and psychiatric diseases [35]. Risk factors can be found on an individual pre-migration level (exposure to war-related trauma, female gender), on a post-migration family level (parental mental health problems and impaired parenting) [36], or as a result of deficient social integration [37].

The rising number of forcibly displaced people and the increased prevalence of mental health disorders have created an urgent demand for psychotherapeutic care that cannot be adequately met. Challenges faced by physicians in the host country—such as limited awareness of asylum seekers’ specific health needs, language and intercultural communication barriers, and difficulties in facilitating their access to and integration into the healthcare system [38]—also affect psychiatric care and psychotherapy. Yet there still exist inequalities between non-immigrants and immigrants in healthcare services [39], reinforced by cultural and structural barriers [40], as well as the negative impact of stigmatization in Arabic [41] and African countries [42].

It is not surprising that the demand for psychotherapy significantly exceeds the supply. For example, psychosocial care centres in Germany were only able to cover 3.1% of the potential demand in 2022 [43]. Untreated disorders not only affect the individuals suffering from them. In refugee populations, children of parents with PTSD and other psychological symptoms show an increased risk of mental health disorders, even if these children have not experienced trauma themselves [44]. The negative consequences for the offspring of traumatized parents include PTSD, anxiety disorders, and an increased risk of abuse and neglect [45].

Given the high prevalence of mental health conditions such as PTSD and depression among refugees, research on attachment patterns in this group is particularly significant. Attachment research can not only contribute to understanding psychological distress in refugees but also provide crucial insights for intervention strategies. This is especially relevant when considering the high prevalence of “unresolved” attachment patterns across nearly all clinical disorders, particularly in PTSD [13].

### 1.3. Attachment Research in Refugees

When studying attachment in refugees, it is essential to consider the cultural and geographical background of their country of origin. Child attachment deviates as follows on a global level: 51.6% secure, 14.7% avoidant, 10.2% resistant, and 23.5% disorganized [46]. Regarding adult attachment, Bakermans-Kranenburg et al. (2024) state that most attachment studies have been conducted in North America and Europe [13]. In Europe, the distribution of adult attachment styles is as follows: Dismissing (Ds) = 20%, Secure (F) = 55%, Preoccupied (E) = 9%, and Unresolved (U) = 15%. In their comprehensive analysis of all attachment representations collected using the Adult Attachment Interview, AAI (George et al., 1985 [15]), they provide the following attachment distribution for the Middle East: D = 30%, F = 46%, E = 6%, and U = 18%. Compared to Europe, this suggests a higher proportion of insecure and unresolved attachment types. Three of the five largest groups of asylum seekers in Europe also come from this region, including Afghanistan, Syria, and Turkey [27]. Unfortunately, no attachment data are available for Middle Eastern clinical groups. However, data from a Western clinical population with PTSD show the following deviation: Dismissing (D) = 10%, Secure (F) = 7%, Preoccupied (E) = 7%, and Unresolved (U) = 76% [13].

There is much less evidence for attachment data outside North America and Europe. One review found that the deviation of attachment patterns, measured in African countries, is similar to the global distribution [47]. A study that measured attachment styles in Turkish mothers found results showing similarities with global results but used a small sample size [48].

To our knowledge, attachment data from clinical samples from the Middle East or other refugee-originating regions are very rare. One Turkish study that explored connections between attachment and post-traumatic growth (PTG) in a non-clinical population found no direct connections with secure attachment but found them with anxious attachment [49], while in a South African student sample, anxious attachment was found to be a negative predictor for PTG [50]. Given the high prevalence of mental disorders among refugees, it is crucial to examine whether the attachment patterns found in Western clinical samples also apply to these populations. The primary causes of forced displacement include conflicts, violence, human rights violations, persecution based on identity or political beliefs, and severe restrictions on public order [51]. These adverse living conditions likely contribute to an increased risk of mental disorders, as reflected in their high prevalence rates. A significant factor in the refugee context appears to be post-migration stress, which has been associated with attachment insecurity. Additionally, higher attachment anxiety has been linked to more severe PTSD symptoms [52].

Considering the overrepresentation of insecure attachment patterns (with 40% “unresolved”) in Western clinical populations [13] in the context of the high prevalence of mental disorders among refugees, it is reasonable to assume that insecure attachment patterns are also highly prevalent in this group. However, research on attachment distribution in refugees remains fragmented. Existing findings on the role of attachment in mental health disorders suggest that attachment patterns play a central role among refugees, a particularly vulnerable population.

The aim of this review is to systematically present the current state of research in answering the following research question: What are the connections between attachment narratives and psychological distress/disorders in refugees?

## 2. Methods

This systematic review follows the methodological and reporting standards outlined in the PRISMA 2020 statement [5] and was registered with PROSPERO on 15 October 2024, under the ID CRD42024590759. The first review protocol is available online at https://www.crd.york.ac.uk/PROSPERO/view/CRD42024590759 (accessed on 8 April 2025).

### 2.1. Eligibility Criteria

Studies were included in this review if they met the following criteria: (1) they examined the relationship between attachment and psychological distress (e.g., trauma, depression, anxiety disorders,…) in refugee populations; (2) they were published within the last 20 years (from 2004 onwards); (3) the study methodology included qualitative, quantitative, or mixed-method approaches, as long as empirical data were collected; (4) the articles were peer-reviewed and published in scientific journals; and (5) the publications were written in English.

Studies were excluded if they (1) did not focus on refugee populations (e.g., studies on voluntarily migrated individuals); (2) did not explicitly examine both attachment and psychological distress; (3) did not present primary or empirical data (e.g., purely theoretical articles); (4) were not peer-reviewed (e.g., conference abstracts, dissertations); or part of grey literature; and (5) were not written in English.

### 2.2. Information Sources and Search Strategy

To identify relevant studies, a systematic literature search was conducted in the databases PubMed, PsycINFO, and Web of Science. The first search was conducted on 20 August 2024, using the following search string: (“attachment”) AND (“refugees” OR “immigrants” OR “asylum seekers” OR “displaced persons” OR “foreigners”). No filters were applied. The second search was conducted on 5 October 2024, using the following expanded search string: (“attachment*” OR “bond*”) AND (“refugee*” OR “asylum seeker*” OR “migrant*” OR “forced migrant*” OR “fleeing migrant*” OR “involuntary migrant*” OR “exile*” OR “displaced person*” OR “foreigner*”) AND (“mental health*” OR “psychological well-being” OR “mental well-being” OR “psychological health” OR “PTSD” OR “posttraumatic*” OR “depress*” OR “anxiety*”). Filters applied: publication date from 2004 to 2024; language = English. All identified records (*n* = 2.951) were imported into a reference management system, and duplicates were removed prior to screening. The screening process followed the PRISMA Guidelines [5].

### 2.3. Screening and Selection of Studies

The screening process consisted of three phases: (1) Title Screening: Articles that were clearly irrelevant were excluded, such as studies on animals or cases where search terms were used in unrelated contexts (e.g., “cell migration”). (2) Abstract Screening: The relevance of each study was assessed based on its abstract. The most common reasons for exclusion at this stage were non-refugee populations, ineligible study designs (e.g., intervention studies without an attachment focus), or incorrect publication types. (3) Full-Text Analysis: Studies were fully reviewed to ensure they met the inclusion and exclusion criteria.

For each included study, data extraction was carried out independently by two reviewers. The extracted data included study characteristics, methods, sample details, measures used, and main findings regarding attachment and psychological distress. The results were then compared, and discrepancies in interpretation or weighting were discussed until a consensus was reached. No automation tools were used, and no contact with study authors was necessary to clarify or obtain missing information.

Data were extracted on the main outcomes of interest, which included any indicators of attachment and psychological distress or mental disorders. Attachment had to be assessed in the psychological sense, referring to the emotional bond between individuals and caregivers or close others, which leads to the formation of an inner working model of attachment [53,54,55]. This included both categorical approaches (e.g., secure, insecure, disorganized attachment patterns or representations) and dimensional instruments (e.g., self-reported scales measuring anxious or avoidant attachment tendencies). The latter ones are based on the social psychological approach of attachment, connected to romantic relationships [56]. Uses of the term “attachment” in other contexts (e.g., attachment to country, culture, or community) were excluded. Measures of mental health were accepted as long as they were clearly situated in a psychiatric or psychological framework, including symptoms or diagnoses of PTSD, depression, anxiety, or general psychological distress. The only restriction for the assessment of mental health was that it had to be examined using a separate instrument, such as a standardized psychological scale or diagnostic tool. This was to ensure that both attachment and psychological distress were assessed as distinct constructs, rather than inferred from a single measure or interview. In addition to outcome data, the following study-level variables were extracted when available: study design, sample size, age group (children vs. adolescents/adults), country of study, setting (e.g., therapy centres, refugee camps), and the instruments used to assess attachment and mental health. Only studies involving refugees, defined as individuals who did not migrate voluntarily, were included. If it was not explicitly clear that the migration occurred involuntarily due to persecution, war, or other threats, the study was excluded. There were no predefined rules for prioritizing specific outcome measures or time points; instead, all relevant findings reported in the included studies were considered. If information was unclear or missing, decisions were made based on consensus between two reviewers. No imputation of missing data was performed.

### 2.4. Assessment of Study Quality

The risk of bias in the included studies was assessed using validated critical appraisal tools, selected according to study design. For qualitative components, the CASP Qualitative Checklist [57] was applied. For quantitative or mixed-method studies with cross-sectional designs, the JBI Checklist for Analytical Cross-Sectional Studies [58] was used. In one case study, the JBI Checklist for Case Reports [59] was applied. All assessments were conducted independently by two authors. Discrepancies in ratings were resolved through discussion and consensus. No automation tools were used in the risk of bias assessment process. Each of these instruments consists of a set of critical appraisal questions: The CASP checklist contains 10 questions, while both JBI checklists contain 8 questions each. CASP items were rated as “Yes”, “No”, or “Not applicable”, whereas JBI checklists included the additional option “Unclear”. To ensure comparability across tools, the number of “Yes” responses for each checklist was calculated as a percentage of the total applicable questions. For studies with a mixed-method design, in which two checklists were applied, the resulting percentages were averaged.

### 2.5. Reporting of Results and Synthesis Methods

No standardized effect measures were used across studies due to the heterogeneity of designs and outcome variables, and no data transformation or statistical conversions were performed prior to synthesis. For quantitative studies, results were reported as stated in the original articles, with a focus on whether statistical tests (e.g., correlations, group comparisons, regressions) yielded significant or non-significant results regarding the relationship between attachment and psychological distress. Exact effect sizes (e.g., coefficients, means) were not extracted systematically.

For qualitative studies, no statistical effect measures were applicable. Findings were reported based on thematic content related to attachment and mental health. Results from qualitative and quantitative studies were compared descriptively and synthesized narratively.

The synthesis focused on identifying the most frequently reported patterns across all included studies. To structure the synthesis, studies were initially grouped by age group (adults/adolescents vs. children) due to differences in the assessment instruments of both attachment and mental health between children and adults.

Results from the included studies were summarized using two structured tables. The first table provided an overview of each study’s authors, title and DOI, instruments used to assess attachment and mental health, as well as the study’s objective. The second table detailed the sample characteristics, including author(s), number of participants, country of origin, mean age, gender distribution, and a summary of the main findings. These tables supported a structured comparison across studies and were supplemented by a narrative synthesis in the text to highlight recurring patterns and methodological differences. Key sources of variation also included measurement approaches (self-report vs. observational/interview-based), study design (qualitative, quantitative, mixed methods), the relationship between informants and constructs (e.g., whether attachment and psychological distress were assessed in the same individual or across different respondents), and slight differences in the operationalizations of attachment. These factors were considered during the synthesis to contextualize differences in findings.

No formal statistical analyses were conducted to explore heterogeneity. However, heterogeneity across studies was examined conceptually and methodologically. No formal sensitivity or subgroup analyses were conducted, but the methodological quality of each study was considered during interpretation, and recurring patterns were evaluated in light of study design and risk of bias to ensure robustness of the synthesis. No formal statistical assessment of reporting bias (e.g., funnel plots or regression-based tests) was conducted due to the narrative nature of the synthesis and the heterogeneity of study designs. However, inconsistencies and non-significant findings were deliberately included and analysed to avoid selective emphasis on positive results. This approach was intended to provide a more balanced understanding of the evidence base.

No formal tool was used to assess certainty. Instead, study quality, consistency of findings, and the relevance of measures were considered when interpreting results.

During the review process, several amendments were made to the original protocol. These included the replacement of MEDLINE with Web of Science to reduce redundancy with PubMed and expand interdisciplinary coverage, refinement of the definition of “refugee” to emphasize involuntary migration, and an adaptation of the synthesis strategy due to methodological heterogeneity, which led to a narrative synthesis rather than a meta-analysis. All deviations are documented and transparently reported in the manuscript.

## 3. Results

At first, we present an overview of the selection process and present descriptive characteristics of the included studies, followed by a summary of the most frequent patterns between attachment and mental health, as well as an overview of the results from a methodological perspective. Finally, inconsistent results will be presented.

### 3.1. Selected Studies and Synthesis of the Results

A total of 2.951 records were identified through database searches: PubMed (*n* = 560), PsycINFO (*n* = 849), and Web of Science (*n* = 1.542). After removing 915 duplicate records, 2.036 records remained for title screening. Of these, 1.890 records were excluded based on title. The remaining 146 records were screened at the abstract level and retrieved for potential full-text inclusion. During this more precise analysis of the inclusion and exclusion criteria, studies that supposedly met all inclusion criteria during the abstract screening were also excluded. For example, the study by Santa-Maria and Cornille [60] was excluded because it did not clearly establish whether the included Latin American immigrants had migrated involuntarily due to persecution, war, or other coercive factors. Although the study addressed attachment and traumatic stress, the refugee status or forced displacement of participants remained unclear. Another example was the study of de Haene et al. [61], which was excluded because it did not include a direct assessment of psychological distress or mental disorder. Although the authors refer to “traumatized refugees” and explored attachment through the Adult Attachment Interview (AAI), mental health outcomes were not assessed using a separate, standardized psychiatric or psychological instrument, and thus did not meet the inclusion criteria.

**Figure 1:**
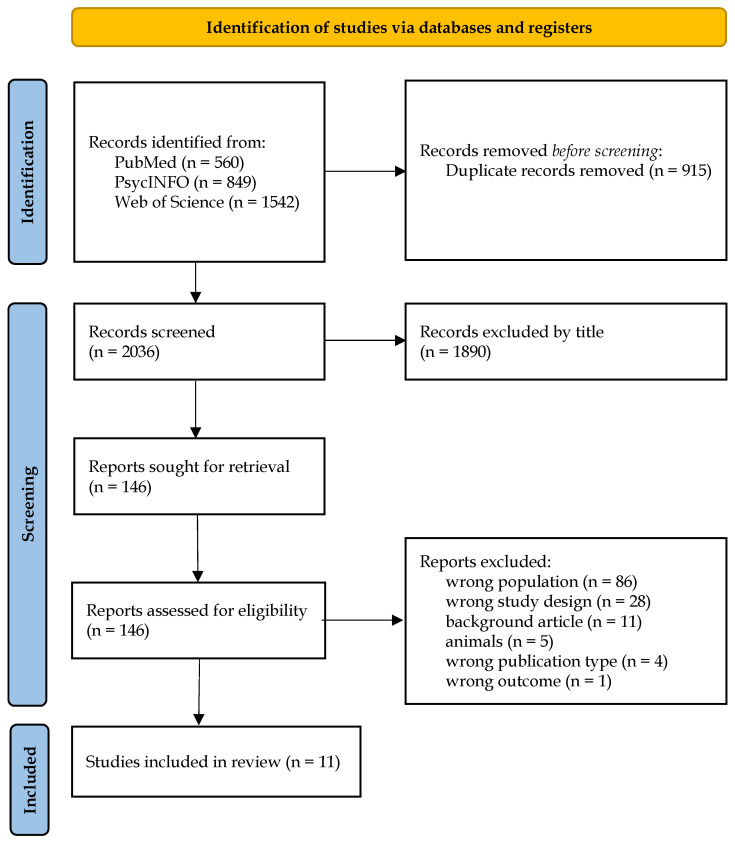
PRISMA flowchart of review progression.

After full eligibility assessment, 135 reports were excluded for the following reasons: wrong population (*n* = 86), wrong study design (*n* = 28), background article (*n* = 11), animal studies (*n* = 5), wrong publication type (*n* = 4), wrong outcome (*n* = 1). In total, 11 studies met the inclusion criteria and were included in the review. The selection process is presented in the PRISMA flow diagram (Figure 1). The risk of bias for each included study was assessed using standardized checklists appropriate to the study design (CASP, JBI cross-sectional, or JBI case report). The results of these assessments, including the percentage of “Yes” responses for each checklist, are presented in Table 1. Methodological quality ranged from 60% to 100%, with an average of 89.66%, indicating generally high study quality. The main characteristics of the included studies (*n* = 11) are summarized in Table 2. This includes information on authorship, publication details, instruments used to assess attachment and psychological distress, and study objectives. Table 3 provides a structured comparison of the most frequently observed result patterns, organized by thematic groupings such as overall trends, population type, methodological approach, and inconsistent findings. The main results of each included study are summarized in Table 4. This includes sample demographics (number of participants, country of origin, age, gender) and main findings. Significant and non-significant results were reported as described in the original publications.

No statistical synthesis or meta-analysis was conducted due to the heterogeneity of study designs, measures, and outcomes. Findings were synthesized narratively and compared between studies. Recurring patterns and inconsistencies were interpreted in the context of study design, type of attachment and mental health assessment, and overall study quality. No sensitivity analyses were conducted. No formal assessment of reporting bias was conducted. However, the synthesis deliberately included and described non-significant and inconsistent findings to reduce the risk of selective reporting and to ensure a balanced interpretation of the evidence.

No formal assessment of certainty was conducted. However, confidence in the body of evidence was judged based on methodological quality, the consistency of findings across studies, and the relevance of study designs and measures to the research question.

The 11 studies were initially grouped by participant age (adults, adolescents, and children). During the synthesis, it became evident that this age-based grouping also reflected a consistent methodological difference: All studies involving adults or adolescents used a within-subject design, assessing attachment and psychological distress in the same individual (*n* = 7), while studies involving children (*n* = 4) typically employed a between-subject design, assessing attachment in the child and psychological distress via caregiver report.

**Table 1:** Methodological quality of the included studies.

Author(s)	Reporting Style	Design Type	Checklist Used	“Yes”-Responses in %
Adults				
[62]	mixed	mixed	CASP; JBI (CS)	100
[63]	self-reporting	quantitative	JBI (CS)	100
[64]	mixed	quantitative	JBI (CS)	100
[65]	non-self-reporting	qualitative	CASP	60
[66]	mixed	quantitative	JBI (CS)	100
Children				
[67]	mixed	mixed	CASP; JBI (CS)	77.50
[68]	mixed	mixed	CASP; JBI (CS)	95%
[69]	self-reporting	quantitative	JBI (CS)	100
[70]	mixed	quantitative	JBI (CS)	100
Adolescents				
[71]	mixed	qualitative	JBI (CR)	75
[72]	mixed	mixed	CASP; JBI (CS)	78.75

Legend—CASP = Critical Appraisal Skills Programme (Qualitative Studies Checklist); JBI (CS) = Joanna Briggs Institute Checklist for Cross-Sectional Studies; JBI (CR) = Joanna Briggs Institute Checklist for Case Reports.

### 3.2. Descriptive Results

Out of eleven included studies, all were published in English. Two publications were conducted in North America, with one in the USA [72] and one in Canada [70]. Seven studies took place in Europe. with three in Denmark [62,65,67], two in the Netherlands [64,68], and one each in Switzerland [63] and Austria [71], and one each in Australia [66] and Tanzania [69]. All articles were published in peer-reviewed journals. For details on study demographics, see Table 2. A comparative summary of grouped results is presented in Table 3; individual study results are listed in Table 4. The most common region of origin (even though not all studies specified the proportion of participants from each region) was the Middle East: Nine out of eleven studies included participants from at least one country in this region. African countries were also frequently mentioned in six out of eleven studies.

Five studies investigated adults, four children, and two adolescents, with one of the latter being a case study. The sample size for adult participants ranged between 43 and 134. The mean age ranged between 30.9 and 42.4 years (Riber, 2016, 2017 did not report participant ages [62,65]). The sample size for child participants ranged between 30 and 226. Three of these studies also examined parents, while one focused solely on mothers. The mean age of the children ranged between 2.5 and 12.1 years, and that of the parents between 29.7 and 41.5 years (Dalgaard et al., 2016 did not report parental ages [67]). In the two studies with adolescent participants, the mean age in the study of Bettmann and Olson-Morrison [72] was 15.9. The participant in the case study [71] was 15 years old.

In the five studies with adult participants, the gender distribution showed a male predominance, with a higher number of male participants in four out of five studies, whereas the gender distribution in the four studies with child participants was similar, with more boys in three out of four studies. The adolescent sample (excluding the single-case study) had a higher number of female participants.

All studies used standardized instruments to measure attachment and psychological disorders/distress, except for one study, which used the *Trauma Coding Manual (TCM)*, a self-developed manual to assess trauma [65]. Attachment was always measured with one single instrument. In total, eight different instruments were used, with the Adult Attachment Interview (AAI) being the only one that was used three times. Mental health problems were measured using 15 different instruments overall, with five studies using more than one instrument. Trauma was the most commonly assessed symptom, with ten out of eleven studies using at least one instrument focused on trauma/PTSD. Among the six studies that exclusively used one instrument, all measured trauma or PTSD. The second most commonly assessed symptoms were depression and anxiety, each examined in four studies. The Harvard Trauma Questionnaire (HTQ) was the most common instrument across all studies, being used in eight different papers. The only other instruments used more than once were the Hopkins Symptom Checklist (HSCL) and Strengths and Difficulties Questionnaire (SDQ). They appeared in two studies.

Overall, two studies only used self-reporting [63,69], and one study solely non-self-reporting instruments [65] The remaining eight studies used mixed approaches. Five studies exclusively used a quantitative approach [63,64,66,69,70]. Four studies used a mixed methods design. In all of these, the qualitative instrument was used to measure attachment [62,67,68,72]. Two studies used a qualitative design: Riber [65], who analysed trauma narratives in refugees, and Gander et al. [71], who employed a single-case study design.

**Table 2:** Summary of included studies.

Authors and Date		Instrument(s)		
	Title and DOI	ATT	MH	Objective
Adults				
[62]	Attachment organization in Arabic-speaking refugees with posttraumatic stress disorder (DOI: 10.1080/14616734.2015.1124442)	AAI	HTQ	To investigate attachment patterns in Arabic-speaking refugees with PTSD, focusing on insecure and unresolved classifications while considering cultural norms and trauma history.
[63]	Attachment style and interpersonal trauma in refugees (DOI: 10.1177/0004867416631432)	ECR	HTQ	To examine whether interpersonal trauma, such as torture, is linked to avoidant attachment and if non-interpersonal trauma affects attachment styles.
[64]	Attachment Representation and Sensitivity: The Moderating Role of Posttraumatic Stress Disorder in a Refugee Sample (DOI: 10.1111/famp.12228)	SBS	HTQ	To analyse the connection between parental attachment representations and sensitivity, exploring PTSD symptoms as a moderating factor.
[65]	Trauma complexity and child abuse: A qualitative study of attachment narratives in adult refugees with PTSD (DOI: 10.1177/1363461517737198)	AAI	TCM	To explore how childhood maltreatment relates to attachment insecurity, relational difficulties, and engagement in psychotherapy among adult refugees with PTSD.
[66]	Activating the attachment system modulates neural responses to threat in refugees with PTSD (DOI: 10.1093/scan/nsab077)	ECR	HTQ, PSS-I, HSCL, PG-13	To examine how attachment system activation influences neural responses to threat in refugees with PTSD, focusing on fear processing and emotional regulation in the amygdala, ventromedial prefrontal cortex, and hippocampus. It also explores the role of insecure attachment and separation experiences in neural dysregulation.
Children				
[67]	The transmission of trauma in refugee families: associations between intra-family trauma communication style, children’s attachment security and psychosocial adjustment (DOI: 10.1080/14616734.2015.1113305)	ATST	SDQ, HTQ, HSCL	To explore how parental trauma history and communication styles relate to children’s attachment security and psychosocial adjustment.
[68]	Parental PTSD, adverse parenting and child attachment in a refugee sample (DOI: 10.1080/14616734.2016.1148748	SSP	HTQ	To examine how parental PTSD symptoms, particularly intrusion and avoidance, relate to child attachment security. It also explores the role of adverse parenting and family-level factors such as composition, child sex, and residency status.
[69])	Fuel to the fire: The escalating interplay of attachment and maltreatment in the transgenerational transmission of psychopathology in families living in refugee camps (DOI: 10.1017/S0954579420000516)	PIML	CTSPC, UCLA RI-5, SDQ, PCL-5, BSI-18	To explore the relationship between parental psychopathology, child attachment, and maltreatment, highlighting stronger links for maternal psychopathology. It explores whether insecure attachment mediates the impact of parental symptoms on child outcomes and how recurrent maltreatment reinforces negative caregiver and self-perceptions.
[70]	Examining Associations between Maternal Trauma, Child Attachment Security, and Child Behaviours in Refugee Families (DOI: 10.25071/1920-7336.41085)	AQS	HTQ, BDI-II, CBCL	To find out how maternal PTSD symptoms correlate with child attachment security and internalizing and externalizing problems, considering attachment security as a moderating factor.
Adolescents				
[71]	Use of the Adult Attachment Projective Picture System in the formulation of a case of an adolescent refugee with PTSD (DOI: 10.1080/15299732.2018.1451803)	AAP	SCID-I, SCID-II, YSR	To explore how the Adult Attachment Projective Picture System (AAP) applies to assess attachment in an adolescent refugee with complex PTSD, focusing on defensive strategies and emotional regulation.
[72]	The relationship between adolescent refugees’ attachment patterns and their experiences of trauma (DOI: 10.1080/15313204.2018.1555499)	AAI	HTQ	To investigate attachment patterns in adolescent refugees and their relationship to trauma, assuming that secure attachment in adolescents is connected with more resilience.

Legend—ATT = Attachment; MH = mental health; AAI = Adult Attachment Interview; HTQ = Harvard Trauma Questionnaire; ECR = Experiences in Close Relationships Questionnaire; SBS = Secure base scripts; TCM = Trauma Coding Manual; PSS-I = PTSD Symptom Scale-Interview; HSCL = Hopkins Symptom Checklist; PG-13 = Prolonged-Grief.

### 3.3. Summarized Results

To answer the research question on the connections between attachment and psychological distress/disorders in refugees, we present the results, beginning with the most frequent patterns.

**Table 3:** Results compared by differentiation criterion.

Criterion	Links/Correlations ^a^	Number of Studies ^b^
Overall Patterns		
Insecure/Unresolved Attachment	Negative psychological outcomes (PTSD, distress, and dysfunctional parenting)	7 [62,63,64,66,67,68,69]
Insecure Attachment/Low attachment Security	Destructive parental characteristics	5 [64,67,68,69,70]
Secure Attachment	Fewer externalizing problems in children, higher parental sensitivity, less impact of maternal PTSD	3 [64,67,70]
By Population		
Adults/Adolescents	Insecure/unresolved attachment and impaired mental health	4 [62,63,64,66]
Children	Insecure attachment and destructive parental characteristics	4 [67,68,69,70]
By Method		
Self-report	Negative effects of insecure attachment on trauma and (maternal) psychopathology	2 [63,69]
Mixed Methods	Insecure/unresolved attachment and negative psychological outcomes	5 [62,64,66,67,68]
Non-self-report	No significant result (descriptive study)	1 [65]
Inconsistent Findings		
Attachment and PTSD	No direct links	4 [63,64,67,70]
Unresolved Attachment vs. PTSD	No significant connection between the “unresolved” category and PTSD symptomatology	1 [62]
Dismissing Attachment and Symptom Levels	No PTSD difference between attachment types; incongruence in trauma reports	[72]

Legend— ^a^ = Unless stated otherwise, reported correlations/links are positive; ^b^ = Total studies reviewed = 11.

Seven of eight studies identified a significant link between insecure attachment (including unresolved patterns) and negative psychological outcomes such as PTSD, distress, and dysfunctional parenting: Insecure attachment correlated with lower parental sensitivity, particularly in parents with high PTSD symptom levels [64]. The “unresolved” attachment classification was significantly more frequent in refugees compared to non-refugee trauma samples [62]. Avoidant attachment was particularly associated with interpersonal trauma [63], while insecure or unresolved attachment correlated with brain activity patterns linked to trauma-processing difficulties [66]. Studies on parenting found that insecure and disorganized attachment in children were linked with parental PTSD symptoms [68]. Insecure attachment was also associated with unfiltered trauma communication [67] and maternal psychopathology [69].

Additional studies provide further context: Gander et al. (single-case study) found that the participant exhibited unresolved attachment along with a PTSD diagnosis [71]. Riber reported high levels of PTSD, attachment insecurity, and unresolved trauma but did not provide specific figures on the exact prevalence [65].

In five studies, the second most frequent result was the role of destructive parental characteristics, including symptomatic stress, with direct [67,68,69] and indirect [64] connections (via PTSD) to insecure attachment, as well as low attachment security in children being a moderator between maternal PTSD and externalizing behaviours in children [70].

Three studies found positive effects of secure attachment: It was demonstrated that secure attachment correlated with fewer externalizing problems in children [67] and higher parental sensitivity [64]. It was also shown that secure attachment moderated the impact of maternal PTSD on child behavioral problems, with securely attached children being less affected [70].

If adults/adolescents and children are analyzed seperately, within the first group, the main pattern still shows a strong connection between insecure/unresolved attachment and impaired mental health [62,63,64,66]. Focusing on the child group, destructive parental characteristics [67,68,69,70] remain the main patterns. Two (child) studies [69,70] additionally conducted mental health assessments in children as well. Focusing on connections within the child group, attachment security did not correlate with child psychopathology in Scharpf and Mkinga et al. [69], but did negatively correlate with internalizing and externalising problems in Barnes and Theule [70].

If results were compared by the method of data reporting, self-report studies (*n* = 2) both showed significant negative effects of insecure attachment on trauma and (maternal) psychopathology [63,69]. The non-self-reporting study (*n* = 1) did not produce statistically significant results, as it was descriptive [65]. Studies with mixed reporting approaches (*n* = 8) showed mostly significant results, except [72] and Gander, Diamond [71], with the first conducting only descriptive analyses and the second being a case study. The most common effect in this group was the same as the most frequent effect, showing connections between insecure/unresolved attachment and negative psychological outcomes [62,64,66,67,68]. Comparing quantitative (*n* = 5), mixed (*n* = 4) and qualitative methods (*n* = 2), there is no new information on the content level. Rather, the qualitative studies confirm, although without significance, the results mentioned above by showing that the category “unresolved” and PTSD often occur together [65,71].

Finally, we focused on possible patterns regarding non-significant and inconsistent results. One pattern could be found in the fact that attachment and PTSD severity seemed to be not always directly linked. Barnes and Theule [70] could not find a direct correlation between maternal PTSD symptoms and child attachment security, nor could Dalgaard et al. [67], who could not find a link between parental PTSD, anxiety, or depression and attachment security in their children. It was also shown that the degree of attachment security was not significantly associated with symptoms of PTSD [64], and interpersonal trauma did not predict anxious attachment [63]. In terms of the deviation in the concrete attachment patterns, no significant connection was found between the “unresolved” category and PTSD symptomatology [62]. Bettmann and Olson-Morrison [72] reported that 46% of adolescents in their sample had an insecure-dismissing attachment style, while 49% were securely attached. Many participants reported different traumatic experiences in the AAI and HTQ, with dismissing individuals showing the most incongruence. However, PTSD symptom levels did not differ significantly between attachment styles.

**Table 4:** Main findings of the included studies.

Authors and Date	*n*	Participant’s Country of Origin	Mean Age	Gender (m/f)	Main Findings
Adults					
[62]	43	Middle Eastern Countries	n.g.	22/21	Most participants displayed insecure attachment patterns (secure: 2.3%, dismissing: 18.6%, preoccupied: 9.3%, and 69.8% were classified as unresolved), a significantly higher proportion than in non-refugee trauma groups. PTSD severity was very high among every attachment group, and, therefore, did not significantly differ across these categories.
[63]	134	Turkey (43% Kurdish), Iran, Sri Lanka, Bosnia, Iraq, Afghanistan, and others (14.9%)	42.4	105/29	Avoidant attachment was significantly associated with interpersonal trauma, such as torture and persecution, while non-interpersonal trauma showed no effect on attachment styles. Anxious attachment was not predicted by either trauma type, supporting the idea that trauma exposure alone may not explain variations in anxious attachment.
[64]	53	Africa, Middle East, South and Eastern Asia, Eastern Europe and Balkan, Russia and former Russian states, South America	30.9	23/30	More secure parental attachment representations correlated positively with parental sensitivity, but PTSD symptoms weakened this relationship. The association between attachment and sensitivity was strongest among parents with high PTSD symptom levels, while those with low PTSD symptoms showed no such pattern.
[65]	43	Iraq (60%), Lebanon and Palestinian territories (30%), two participants from other countries in the Arabic region.	n.g.	22/21	Among 43 refugees with PTSD, 27 reported severe childhood abuse, primarily physical, often resembling torture. Trauma related to attachment and war co-occurred from early childhood, contributing to insecure attachment, low self-esteem, relational difficulties, and emotional distress. These vulnerabilities may hinder engagement in psychotherapy due to difficulties with trust and emotional regulation.
[66]	50	Iran, Iraq, Tibet, Afghanistan, Bosnia-Herzegovina, Cambodia, Bhutan, Morocco, Myanmar, Chile, Fiji, Ghana, Kuwait, Laos, Nigeria, Tibet, and Vietnam	40.6	30/20	Activating the attachment system altered neural threat responses in refugees with PTSD, leading to increased amygdala activity and reduced ventromedial prefrontal cortex regulation in those with high separation grief. Avoidant attachment was linked to increased frontoparietal activity, suggesting an attempt to suppress emotions, but left amygdala hyperactivity indicated ineffective regulation. Anxious attachment was associated with reduced dorsomedial prefrontal cortex activity and weaker amygdala-prefrontal connectivity, suggesting heightened emotional responses and impaired regulation.
Children					
[67]	30 ^a^ 60 ^b^	Iraq, Iran, Lebanon, Palestine, Syria and Afghanistan	6.8 ^a^ n.g. ^b^	16/14 ^a^	Refugee children exhibited more psychosocial difficulties than Danish peers. Secure attachment was negatively correlated with externalizing problems, but no significant association was found with internalizing symptoms. Parental PTSD, anxiety, and depression levels were not significantly related to child attachment security. However, intra-family trauma communication played a role, with unfiltered parental speech about trauma being strongly linked to insecure attachment in children.
[68]	50 ^a^ 68 ^b^	Middle East (36.8%), Africa (36.8%), East Europe (14.7%), Asia (7.4%), and South America (1.5%)	2.5 ^a^ 35.5 ^c^ 29.7 ^d^	31 ^a^/19 ^a^ 27 ^c^/41 ^d^	Parental PTSD symptoms, particularly intrusion and avoidance, were strongly associated with child attachment insecurity and disorganization. While adverse parenting behaviours were expected to mediate this relationship, they played a less significant role. The parent-child dyad had a greater influence on attachment security than broader family-level factors like child sex, residency status, or family composition.
[69]	226 ^a^ 452 ^b^	Burundi	12.1 ^a^ 41.5 ^c^ 34.5 ^d^	120/106 ^a^ 226/226 ^b^	Higher levels of maternal—but not paternal—psychopathology were directly linked to increased child maltreatment and insecure attachment. Insecure attachment did not mediate the relationship between parental and child psychopathology. Instead, maltreatment itself was the primary link, reinforcing negative representations of the mother and self, which contributed to child psychopathology.
[70]	36 ^a^, 36 ^d^	Syria (83.3%), two from Palestine, one each from Uganda, Egypt, Nigeria, and Nicaragua	3.7 ^a^ 32.7 ^d^	n.g. ^a^ n.g. ^d^	No direct correlation was found between maternal PTSD symptoms and child attachment security. However, maternal PTSD was strongly associated with child externalizing and internalizing behaviours. Attachment security played a moderating role, with higher security reducing the impact of maternal PTSD on child behavioural problems.
Adolescents					
[71]	1	Iraq	15	1/0	A case study of an adolescent refugee with PTSD classified the individual as having an unresolved attachment pattern. The participant relied on defensive mechanisms such as deactivation and cognitive disconnection in response to distress. These patterns contributed to significant trauma-related symptoms and difficulties with emotional and cognitive regulation, affecting daily functioning.
[72]	37	Somalia, Sudan, Kenya, Liberia, Rwanda, The Republic of Congo, Russia, Guinea, Vietnam, Mexico, Burundi, and Togo.	15.9	16/21	Among adolescent refugees, 48.7% were securely attached, 45.9% dismissing, and only a small proportion were disorganized (2.7%) or preoccupied (2.7%). Given the participants’ exposure to war-related trauma, this distribution was unexpected. PTSD symptom severity did not differ significantly between securely attached and dismissing participants, raising questions about the assumed protective role of secure attachment. Additionally, dismissing individuals reported more inconsistencies between trauma disclosures on the AAI and HTQ, suggesting that attachment style may influence trauma processing and reporting.

Legend—n.g. = not given; ^a^ = child sample; ^b^ = parents; ^c^ = fathers; ^d^ = mothers; AAI = Adult Attachment Interview; HTQ = Harvard Trauma Questionnaire.

## 4. Discussion

This systematic review sought to investigate the connections between attachment and mental health in refugees. The most frequent finding was a link between insecure/unresolved attachment patterns and impaired mental health, including PTSD, general psychopathology, difficulties in neuronal trauma processing, and parental misconduct (when parents suffered from mental health problems). The negative association between insecure attachment and PTSD is in line with previous reviews [2,4,6] and is not surprising, given that the prevalence of PTSD and depression is higher in refugees than in the general population [29,31]. Additionally, children’s PTSD symptom levels are largely associated with their parents’ symptom levels [44].

Regarding the connection between insecure/unresolved attachment and destructive parental characteristics and symptoms, earlier findings support our results as well [25,73]. The third most frequent result was the positive/protective effect of secure attachment in children with parents suffering from trauma and psychopathological problems. This aligns with Cooke et al. [74], who found that securely attached children experienced more positive affect, less negative affect, better emotion regulation, and more frequent use of cognitive and social support coping strategies. The positive effects of secure attachment in children are also significantly and positively related to parental responsiveness, autonomy support, and behavioural control [75]. This suggests that traumatized refugee parents can still provide the conditions necessary for establishing secure attachment despite facing multifactorial stressors.

When results are reported separately for adults and children, the most common effect in the adult group aligns with the overall most frequent finding, highlighting a strong connection between insecure/unresolved attachment and impaired mental health in refugees. The same was true for results regarding children, where the most common finding was the connection between destructive parental characteristics or symptoms and insecure/unresolved attachment. In this context, caregivers with unprocessed trauma may withdraw or behave aggressively, sending conflicting signals that confuse and distress the child. This inconsistency can disrupt attachment development, leading to unresolved representations marked by trauma or loss. Children in this state face a painful conflict: avoid their caregiver and remain emotionally dysregulated, or approach them despite fear, attempting to care for the caregiver. Without a secure attachment model, they see the world as unsafe and others as unreliable, resulting in poor emotional regulation and an inability to manage inner experiences. The two studies investigating adolescents used sample sizes that were too small to generate meaningful results.

Overall, the results of this review are consistent with and extend existing findings on the interplay between attachment and mental health, especially in trauma-exposed populations.

### 4.1. Results from a Methodological Perspective

Studies using self-reporting (for both attachment and mental health) and mixed-reporting instruments (with two exceptions) consistently found significant results, whereas the one non-self-reporting study did not produce statistically significant findings. Regarding trauma, this aligns with previous meta-analyses showing that the association between attachment and posttraumatic symptoms is stronger when PTSD is measured using self-reporting instruments [2,4]. However, it must be acknowledged that, for instance, evidence regarding the diagnostic accuracy of screening questionnaires for PTSD in Arabic-speaking populations is still lacking [76].

Regarding attachment, distinguishing between self- and non-self-reporting measurement instruments is unnecessary for children, as self-report measures lack validity in this population [77]. However, when focusing on adults and adolescents, self-report instruments appear to yield more significant results. In all three studies that used a self-reporting attachment instrument, significant associations between attachment (measured with the ECR and PIML) and trauma were found [63,66,69]. In contrast, five out of eight studies that measured attachment using non-self-reporting instruments [62,64,68,70,78] produced significant attachment-related results, while the other three [65,71,72] did not.

The high reliability of the ECR has been demonstrated [79]. However, the comparatively lower number of significant results from representational/narrative instruments like the AAI or AAP should not be interpreted as a lack of reliability, as these instruments exhibit strong psychometric properties [16,80,81]. One possible explanation for this discrepancy is that interview-based and self-report methods assess attachment differently. Self-reporting instruments capture a conscious approach to attachment styles, whereas the AAI and AAP assess unconscious processes and states of mind [17]. This could explain why these methods correlate poorly. Attachment is commonly assessed through self-report questionnaires and representational interviews (like the AAI or AAP). Self-report tools differ in theoretical grounding—some focus on romantic “attachment styles” rooted in personality theory, lacking ties to developmental models. Others assess parent–child attachment, aligning more closely with developmental theory, though conceptual definitions vary. In contrast, interviews like the Adult Attachment Interview (AAI), its adolescent adaptations (e.g., CAI) or the Adult Attachment Projective Picture System (AAP) are based on developmental attachment theory, analysing how individuals mentally organize attachment experiences. They capture attachment dimensions and uniquely assesses constructs from Bowlby’s theory, such as defensive processing, trauma-related content, agency of the self, and responses to solitude versus interaction. Furthermore, they allow the classification of the unresolved attachment pattern. They have shown clinical relevance in adults and may offer valuable insight into attachment patterns and their link to psychopathology [82].

In terms of data collection methods, quantitative studies dominate both in number and relevance, although the results of non-quantitative studies support the most frequent findings.

### 4.2. Inconsistencies and Limitations of the Included Studies

The evidence included in this review presents several limitations. Some studies—particularly those involving adolescents—had small sample sizes, limiting generalizability. Most included studies employed cross-sectional designs, which limit the ability to draw causal conclusions. In addition, the relationship between attachment insecurity and trauma-related psychopathology may be bidirectional and dynamic. Traumatic experiences in early childhood appear to disrupt the development of secure attachment more profoundly than those occurring later in life. As a result, they may impair the child’s ability to regulate negative affect and cope with stress in adaptive ways. Deficits in these regulatory capacities, in turn, increase vulnerability to the development of posttraumatic stress disorder [1]. Moreover, trauma-induced changes in attachment representations may influence future parenting behaviour and attachment styles toward one’s own children, potentially contributing to the intergenerational transmission of insecurity [23]. These processes highlight the importance of viewing attachment not only as a predisposing factor for trauma-related disorders but also as a system that may itself be altered by traumatic experiences. Longitudinal research is needed to better understand these reciprocal influences over time.

There was also considerable methodological heterogeneity, with a wide range of instruments used to assess both attachment and psychological distress, complicating direct comparisons across studies. Regarding attachment, the AAI—the most frequently used non-self-report instrument—has been shown to be applicable in the Middle East [13], whereas the ECR—the most frequently used self-report instrument—lacks cross-cultural validation [63]. In terms of mental health assessment, many authors of the included studies noted that the HTQ, the most commonly used instrument, has been validated in refugee populations [62,64,67,68,70]. However, in the child-focused studies, mental health was assessed via caregiver report in two cases, directly in children in one case, and not assessed at all in one study. This inconsistency, along with the reliance on parental reporting, may introduce bias—particularly when caregivers themselves are affected by trauma.

Finally, most instruments used were developed in Western cultural contexts, raising concerns about their cultural appropriateness and validity for diverse refugee populations.

Inconsistent results were observed regarding the relationship between PTSD symptoms and attachment, with both significant and non-significant effects occurring. However, plausible explanations exist for these discrepancies. Bettmann and Olson-Morrison [72], Riber [62], and Dalgaard [67] did not find associations between the “unresolved” attachment category and PTSD severity. In the first study mentioned, it should also be noted that Bettmann and Olson-Morrison [72] did not use inferential statistics and that the mean HTQ scores across all attachment patterns were below the clinical cutoff. Riber [62] explained this finding with a ceiling effect, where PTSD scores were uniformly high across all participants, making differences between attachment groups difficult to detect. Dalgaard [67] reported similarly high scores in PTSD, depression, and anxiety, resulting in limited variance.

Another possible explanation for non-significant effects is the heterogeneity of samples in many studies. For example, three studies reported non-significant correlations between attachment security and trauma while using participants from more than five different home countries and relatively small sample sizes, increasing heterogeneity and reducing the likelihood of finding significant effects [64,67,70].

Considering that most refugees examined in this review originate from Africa and the Middle East, prevalence rates for these regions vary both regionally and across age groups. For example, Steel et al. [83], in their systematic review and meta-analysis, report that one-year prevalence rates for common mental disorders in adults in Sub-Saharan Africa were low. In contrast, Jakobsson et al. [84] found high prevalence rates of depression and PTSD among adolescents in the same region, with a positive correlation between age and prevalence. Rates for mental disorders are generally high in the WHO Eastern Mediterranean Region [85]. Moreover, PTSD and depression are highly prevalent in politically unstable countries (e.g., Libya) [86] and among war survivors still living in conflict regions [87]. Cultural heterogeneity across samples may also contribute to inconsistent results, as the literature on cultural concepts of distress and psychiatric disorders lacks epidemiological rigor [88].

### 4.3. Limitations

Cultural heterogeneity and language barriers always lead to increased complexity in research with refugees. Most of the instruments used to assess attachment and psychological distress were developed in Western contexts. In addition, relevant sample characteristics, such as country of origin, ethnic background, and stage of displacement (e.g., in transit, asylum seeking, resettled), were not always systematically reported, limiting the ability to contextualize findings. One possible reason for this lack of contextual data may be the specific vulnerability of refugee populations in research settings. Many refugees experience chronic insecurity and may be reluctant to disclose personal or migration-related information for fear that it could negatively affect their asylum process. Heightened anxiety, mistrust of institutions, and language barriers can further complicate data collection in this population.

While attachment theory has been shown to describe core features of relational development across cultures, the meaning and expression of attachment behaviours, narratives, and self-reports are influenced by cultural norms and expectations. For example, recent large-scale research analysing more than 26,000 Adult Attachment Interviews across continents found both cultural variation and universal patterns in attachment classification. While the secure pattern was most common across all regions, certain trends, such as higher rates of dismissing attachment in Middle Eastern populations and lower prevalence of unresolved attachment in Western Europe, were observed [13].

These findings suggest that attachment is a biologically rooted but culturally shaped system and that assessment tools must account for this interplay. We therefore recommend that future research on refugee populations prioritize cultural adaptation (e.g., less language-based instruments like the AAP) and validation of instruments, integrating both universalist and culture-sensitive theoretical frameworks.

Cultural differences emphasize the need for adaptations, including adjustments to personnel and setting, modifications to content, and the translation or adaptation of assessment tools [89], as well as more refugee participation, more conceptual clarity, and a better recognition of structural barriers [90]. While language barriers could lead to difficulties in the diagnostic process [91], there is also evidence of the positive impact of interpreters on clinical outcomes [92].

From a methodological perspective, two approaches exist for assessing attachment: using questionnaires or interviews. Ravitz et al. [17] recommend the following in their review: The AAI and the AAP are both established tools to assess attachment representations in adolescents and adults in many settings. When attachment is a secondary focus and conscious evaluations of attachment relationships are of interest, short self-report tools like the Relationship Questionnaire (RQ) offer a practical solution. The authors also suggest that categorical attachment classifications (e.g., secure, insecure, disorganized/unresolved, organized) are especially useful in clinical settings.

An additional definitional problem in research on forced migrants with mental illnesses is the inconsistent interpretation of the term “refugee” across studies. Many studies operationalize this term to include patients from treatment centres for refugees or migrants, residents of community accommodations for asylum seekers, migrants with mental health problems, or individuals living in refugee camps. None of these definitions strictly adhere to the Protocol of the Refugee Convention in 1951/1967 [93], and the lack of consistency in these operational definitions poses significant methodological challenges.

In addition to these conceptual and methodological limitations, the review process itself also has certain constraints. This review was pre-registered in PROSPERO, enhancing its transparency and methodological rigor. However, several deviations from the original protocol occurred. For instance, MEDLINE was replaced with Web of Science to reduce overlap with PubMed, as both databases are largely based on the same source (MEDLINE) and share many indexed articles. Web of Science was chosen instead to allow for broader interdisciplinary coverage, including sources from psychology, social sciences, and humanities. The inclusion criteria were refined during the review process, particularly in terms of operationalizing the term “refugee” as individuals who migrated involuntarily. This refinement contributed to the relatively small number of included studies (*n* = 11). However, given that forced migration often entails a fundamental disruption of one’s sense of safety and continuity, and considering the potential interplay between trauma and attachment, we deemed this criterion essential to maintaining conceptual clarity and relevance in our synthesis.

Additionally, the planned synthesis strategy was adapted in response to the heterogeneity of study designs and outcomes, which made a meta-analytic approach unfeasible and led to a narrative synthesis. Further limitations of the review process include the restriction to English language publications, the use of only three databases, and the exclusion of grey literature, all of which may have resulted in the omission of relevant studies. Although study screening and risk of bias assessment were conducted by two independent reviewers, no third reviewer was involved to resolve disagreements. Despite these adjustments, the review adhered to the core principles of a systematic review. The literature search, study selection, data extraction, and synthesis were conducted in a structured, transparent, and reproducible manner, in line with the PRISMA 2020 guidelines [5].

## 5. Conclusions

This systematic review investigated the role of attachment in refugees suffering from mental illnesses. Across the 11 included studies, PTSD/trauma was the most commonly studied condition. Results were partially reported separately for adults/adolescents and children due to differences in study designs. Insecure attachment (including unresolved attachment representations) showed the strongest associations with negative psychological outcomes such as PTSD, distress, and dysfunctional parenting, particularly within adult samples. The second most common finding was found between insecure/unresolved attachment in children and destructive parental characteristics and symptoms. The third most common effect showed that secure attachment had a protective effect against negative outcomes, including externalizing problems and maternal PTSD symptoms, as well as a positive correlation with parental sensitivity.

In sum, the findings presented in this systematic review suggest a strong connection between insecure and unresolved attachment and psychopathology, as well as other destructive characteristics, while secure attachment serves as a protective factor. These connections between attachment and mental health in refugees follow a similar pattern to those observed in Western (clinical) samples. Non-significant effects between PTSD/trauma and insecure attachment were largely attributed to sample heterogeneity and small sample sizes. The relatively small number of included studies highlights the need for more empirical work that bridges attachment and psychological distress in refugee populations. Expanding this research field—especially with culturally adapted methodologies—could support the development of more targeted clinical interventions.

Future studies could aim for a more homogeneous sample, for instance by limiting the number of countries of origin represented in the study population.

### Clinical Implications

Our results highlight that attachment is a crucial factor in the treatment of refugee patients. These findings are similar to those observed in Western populations. Refugees are highly vulnerable even within clinical samples, as they also face socioeconomic and interpersonal challenges, as well as stressors related to the asylum process and immigration policies [94]. Research on the development of health asset measurement tools for refugees is still in its early stages. Further studies are needed to create individual health asset tools that are suitable for use in multicultural communities, including refugee populations [95]. The same problem applies to children [96]. Regarding trauma, there are numerous challenges and inconsistencies in delivering transcultural, trauma-informed care. Respondents highlighted the importance of comprehensive yet adaptable approaches that accommodate diverse cultural perspectives on mental health [97].

Regarding attachment, treatment engagement differs across attachment patterns, with securely attached individuals showing higher engagement and fearful individuals displaying the lowest engagement [98]. Securely attached individuals also tend to develop stronger therapeutic alliances than those with less secure attachment styles [21]. Given that refugee populations often exhibit heightened mistrust and are more hesitant to seek treatment, understanding attachment representations could help bridge this gap by emphasizing attachment-related interactions in therapeutic settings. One important concept in terms of psychopathology and the psychotherapy process, linked to attachment, is reflective functioning/mentalization [99], which in turn seems to be a mediator of change in psychotherapy [100]. This aligns with the concept of “earned secure attachment”, which may be fostered by developing reflective functioning skills and establishing secondary attachment figures [101]. Regarding children, empirically supported interventions such as Attachment-Based Family Therapy (ABFT) could serve as effective treatment approaches [102] for refugee offspring. Since the vast majority of refugees originate from collectivist cultures, psychotherapists must be mindful of cultural differences in emotional expression. Compared to Western cultures, individuals from collectivist societies tend to prioritize emotional regulation and social harmony over explicit emotional expression [103].

In summary, previous research with refugees shows that this group is comparable to Western samples in terms of the connection between attachment and mental health. However, cultural and linguistic factors must be precisely taken into account.

Future research should aim to investigate attachment in more homogeneous refugee groups—potentially focusing on a single country of origin with a clearly defined sample. Additionally, cultural background should be carefully considered when selecting measurement tools, opting for self-report attachment instruments for capturing conscious attachment styles and interview-based assessments (e.g., AAI and AAP) for capturing unconscious attachment representations and in-depth attachment analysis, with the unique option to classify unresolved attachment representations, which seem to be especially relevant for this specific population.

## Data Availability

No new data were created or analyzed in this study.
